# From Teratoma to Pacemaker: A Rare Case of Anti-N-Methyl-D-Aspartate Receptor (Anti-NMDAR) Encephalitis With Severe Autonomic Instability

**DOI:** 10.7759/cureus.94591

**Published:** 2025-10-14

**Authors:** Mohamad A Bahrou, Samie Gilani, Mohamed R Labedi, Momin Siddique

**Affiliations:** 1 Internal Medicine, Southern Illinois University School of Medicine, Springfield, USA; 2 Cardiology, Southern Illinois University School of Medicine, Springfield, USA

**Keywords:** anti-nmdar encephalitis, asystole, bradyarrhythmia, dysautonomia, pacemaker

## Abstract

We report a rare case of an 18-year-old female patient who presented with fever, altered mental status, and seizures and was diagnosed with anti-N-methyl-D-aspartate receptor (anti-NMDAR) encephalitis in the setting of a right ovarian teratoma with high-titer antibodies. Despite immunotherapy and tumor resection, she developed severe autonomic instability characterized by recurrent bradycardia, sinus pauses, and an episode of asystole, necessitating temporary transvenous pacing followed by permanent pacemaker (Boston Scientific, USA) implantation. This case highlights the potential for life-threatening cardiac complications in anti-NMDAR encephalitis and emphasizes the need for continuous cardiac monitoring, timely intervention, and multidisciplinary management.

## Introduction

Anti-N-methyl-D-aspartate receptor (anti-NMDAR) encephalitis is a rare autoimmune disorder strongly linked to ovarian teratomas, with an incidence of approximately one in 1.5 million annually [1]. Autoantibodies against central nervous system (CNS) NMDARs drive a spectrum of neuropsychiatric and autonomic manifestations. Among these, dysautonomia represents a severe and potentially fatal complication, with profound cardiovascular consequences including bradyarrhythmias, sinus pauses, and asystole [1,2]. We describe a case of anti-NMDAR encephalitis in a young female patient who developed profound dysautonomia, necessitating permanent pacing.

## Case presentation

Initial presentation

An 18-year-old female patient with a history of depression and occasional marijuana use presented to the emergency department with a subacute onset of headache, fever, and altered mental status, characterized by agitation and a manic-like episode beginning earlier the same day. Notably, she had no prior history of manic or psychotic episodes.

Vitals and examination

On admission, the patient was disoriented and confused. Vital signs included a temperature of 38.1°C, blood pressure of 152/100 mmHg, heart rate of 103 bpm, respiratory rate of 18 breaths per minute, and oxygen saturation of 98% on room air. Neurological examination was limited due to her mental status but revealed no focal deficits.

Investigations and clinical course

Initial laboratory workup showed a WBC count of 17.7 × 10³/µL. Urine toxicology was positive for cannabinoids and benzodiazepines. Brain magnetic resonance imaging (MRI) was nondiagnostic due to motion artifact but revealed no acute abnormalities. Given concern for CNS infection, a lumbar puncture was performed. Cerebrospinal fluid (CSF) analysis demonstrated WBCs 95 cells/µL (99% lymphocytes, 1% monocytes), RBCs 30 cells/µL, protein 31 mg/dL, and glucose 73 mg/dL. CSF cultures and polymerase chain reaction (PCR) panel were negative for common bacterial and viral pathogens, including herpes simplex virus type 1, herpes simplex virus type 2, varicella zoster virus, cytomegalovirus, human herpesvirus 6, and enterovirus.

**Figure 1:**
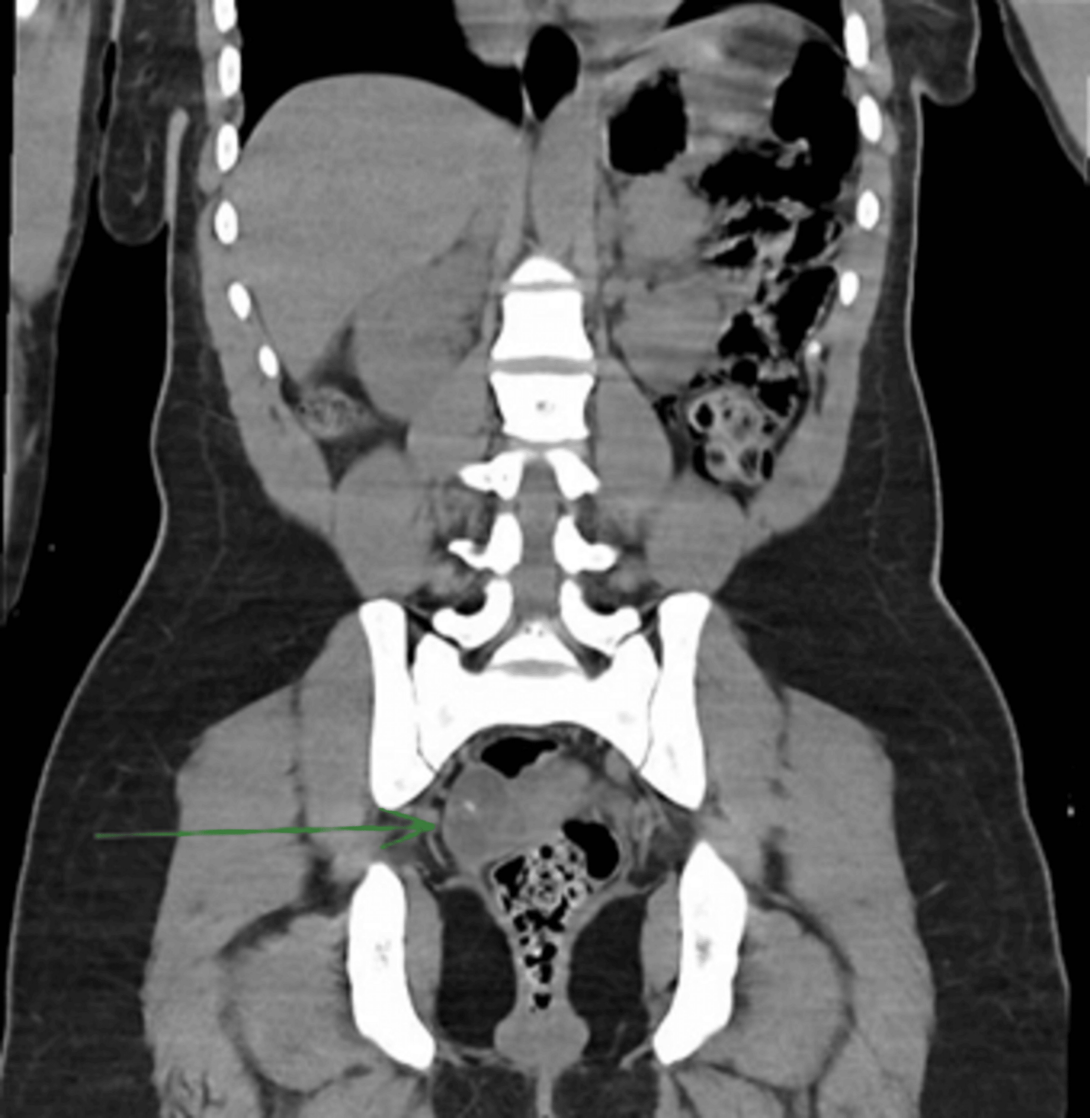
Coronal CT scan showing a 6.1 cm right ovarian teratoma (green arrow). CT: Computed tomography

**Figure 2:**
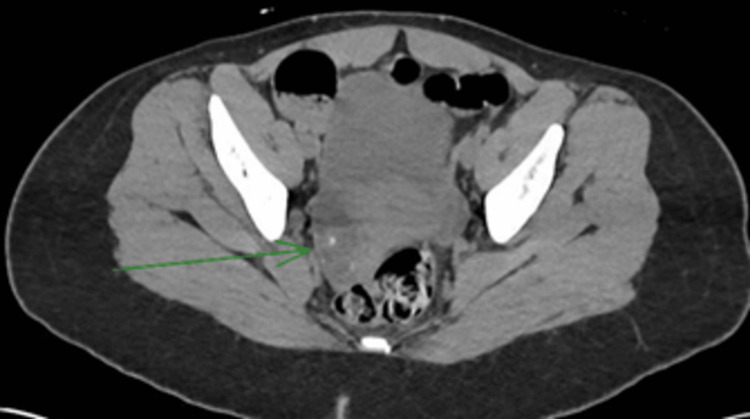
Axial CT scan showing a 6.1 cm right ovarian teratoma (green arrow). CT: Computed tomography

Electroencephalogram (EEG) showed a background rhythm of 3-4 Hz in the theta range, symmetric bilaterally without asymmetry, with no focal, lateralizing, or epileptiform abnormalities - findings consistent with severe diffuse encephalopathy. Computed tomography (CT) of the abdomen and pelvis revealed a 6.1 cm complex right ovarian mass suggestive of a germ cell tumor (Figures 1-2). Over the subsequent days, the patient’s condition deteriorated, with progressive development of dystonia and seizures. In the absence of infectious findings and in the setting of an ovarian mass, an autoimmune etiology was strongly suspected - specifically anti-NMDAR encephalitis.

Autoimmune and paraneoplastic antibody testing in both serum and CSF confirmed high-titer NMDAR antibodies, establishing the diagnosis of anti-NMDAR encephalitis.

**Figure 3:**
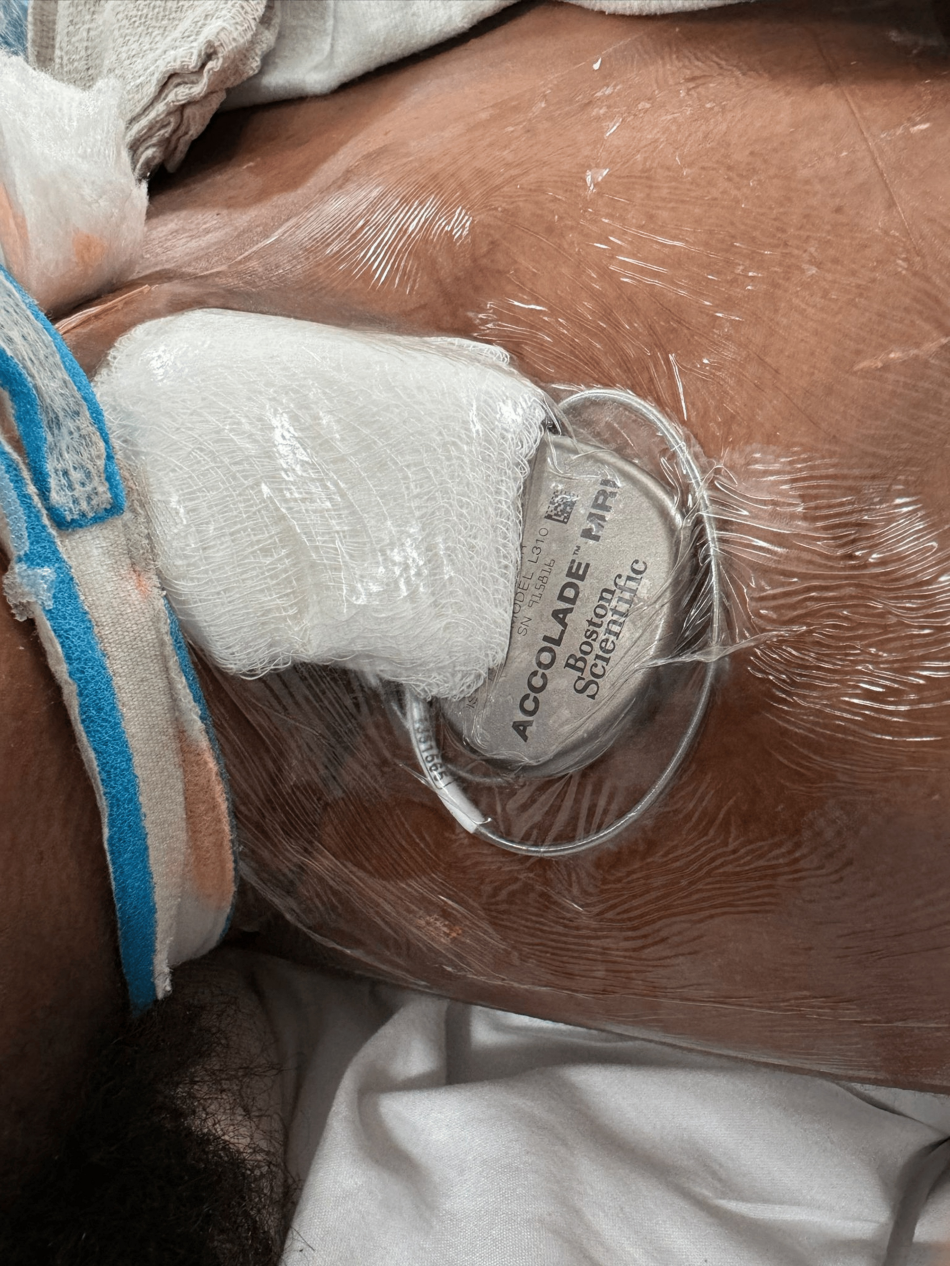
Temporary pacemaker with active-fixation lead in place.

The patient was initiated on aggressive immunotherapy, including high-dose intravenous methylprednisolone, intravenous immunoglobulin (IVIG), and plasmapheresis. She subsequently underwent a right oophorectomy, with pathology confirming a mature ovarian teratoma. Despite these interventions, she developed profound autonomic instability, manifested by episodes of supraventricular tachycardia (heart rate up to 180 bpm), bradycardia (heart rate as low as 30 bpm), labile blood pressure, and prolonged sinus pauses (maximum duration of 30 seconds). She ultimately experienced an episode of asystole, necessitating placement of an active-fixation transvenous temporary pacing lead (Boston Scientific, USA) (Figure 3) and, subsequently, implantation of a single-chamber leadless pacemaker (Boston Scientific, USA).

During her prolonged eight-month ICU hospitalization, the patient demonstrated slow but gradual recovery of both neurological function and autonomic regulation.

## Discussion

This case highlights a severe presentation of anti-NMDAR encephalitis secondary to an ovarian teratoma, complicated by significant autonomic dysfunction. In 2021, a study reported autonomic dysfunction in 61.3% of 119 patients with this condition [3]. Clinical manifestations included tachycardia, bradycardia, hypersalivation, central hypopnea, labile blood pressure, and hyperhidrosis. The exact pathophysiological mechanism remains unclear but is thought to involve damage to autonomic nerve centers and disruption of glutamate neurotransmission by anti-NMDAR antibodies [3,4].

While autonomic instability is a recognized feature of anti-NMDAR encephalitis, the development of life-threatening arrhythmias and asystole requiring pacemaker implantation, as seen in this patient, is rare [5]. In a 2008 case series of 100 patients with anti-NMDAR encephalitis, 37 developed arrhythmias and only four required pacemaker implantations [2]. 

Heart rate regulation is a complex process governed by both the parasympathetic and sympathetic nervous systems, mediated by autonomic centers in the brain. In anti-NMDAR encephalitis, this regulation appears to be disrupted by circulating antibodies [6]. Additionally, seizures may further contribute to autonomic dysregulation. One case report documented a 15-second episode of asystole associated with ictal discharges of temporal origin while the patient was monitored via EEG, highlighting that seizures can precipitate asystole in this setting [7].

In our case, the patient developed severe dysautonomia with symptomatic bradycardia, sinus pauses, and a documented episode of asystole. These events meet a Class I indication for permanent pacemaker implantation as outlined in the 2018 American College of Cardiology/American Heart Association/Heart Rhythm Society (ACC/AHA/HRS) guidelines on the evaluation and management of bradycardia and cardiac conduction delay. The guidelines recommend pacing for patients with symptomatic sinus node dysfunction or asystole not attributable to reversible causes [8]. Due to life-threatening cardiac instability and ongoing seizures, a temporary transvenous pacemaker with active-fixation lead was placed emergently. Because of her persistent high pacing requirements, this was subsequently followed by permanent pacemaker implantation to prevent further autonomic crises.

Patients with dysautonomic manifestations often require ICU management with continuous monitoring due to the risk of rapid clinical deterioration. Effective management of such complex presentations demands a multidisciplinary team approach to ensure optimal outcomes.

## Conclusions

This case underscores the potential for profound autonomic instability in anti-NMDAR encephalitis, including life-threatening bradyarrhythmias and asystole. Early recognition of autonomic dysfunction, close hemodynamic monitoring, and timely pacemaker implantation are critical to prevent fatal outcomes. A multidisciplinary approach involving neurology, cardiology, and intensive care is essential to optimize recovery in these patients.
